# Cardioprotection stimulated by resveratrol and grape products prevents lethal cardiac arrhythmias in an animal model of ischemia and reperfusion

**DOI:** 10.1590/ACB360306

**Published:** 2021-05-07

**Authors:** Francisco Sandro Menezes-Rodrigues, Paolo Ruggero Errante, Erisvaldo Amarante Araújo, Mariana Pontes Pacheco Fernandes, Michele Mendes da Silva, Marcelo Pires-Oliveira, Carla Alessandra Scorza, Fúlvio Alexandre Scorza, Murched Omar Taha, Afonso Caricati-Neto

**Affiliations:** 1 PhD. Universidade Federal de São Paulo – Laboratory of Autonomic and Cardiovascular Pharmacology – São Paulo (SP), Brazil.; 2 Bsc. Universidade Federal de São Paulo – Laboratory of Autonomic and Cardiovascular Pharmacology – São Paulo (SP), Brazil.; 3 Graduate student. Faculdades Oswaldo Cruz – São Paulo (SP), Brazil.; 4 Graduate student. Universidade Paulista – São Paulo (SP), Brazil.; 5 PhD. Assistant Professor. União Metropolitana de Educação e Cultura – School of Medicine – Lauro de Freitas (BA), Brazil.; 6 Associate Professor. Universidade Federal de São Paulo – Department of Neurology and Neurosurgery – São Paulo (SP), Brazil.; 7 Associate Professor. Universidade Federal de São Paulo – Department of Surgery – São Paulo (SP), Brazil.; 8 Associate Professor. Universidade Federal de São Paulo – Department of Pharmacology – São Paulo (SP), Brazil.

**Keywords:** Myocardial ischemia, Reperfusion Injury, Atrioventricular block, Wine, Rats

## Abstract

**Purpose:**

To evaluate the preventive cardioprotective effects of resveratrol and grape products, such as grape juice and red wine, in animal model of cardiac ischemia and reperfusion.

**Methods:**

Male Wistar rats orally pretreated for 21-days with resveratrol and grape products were anesthetized and placed on mechanical ventilation to surgically induce cardiac ischemia and reperfusion by obstruction (ischemia) followed by liberation (reperfusion) of blood circulation in left descending coronary artery. These rats were submitted to the electrocardiogram (ECG) analysis to evaluate the effects of pretreatment with resveratrol and grape products on the incidence of ventricular arrhythmias (VA), atrioventricular block (AVB) and lethality (LET) resulting from cardiac ischemia and reperfusion.

**Results:**

It was observed that the incidence of AVB was significantly lower in rats pretreated with resveratrol (25%), grape juice (37.5%) or red wine (12.5%) than in rats treated with saline solution (80%) or ethanol (80%). Similarly, incidence of LET was also significantly lower in rats pretreated with resveratrol (25%), grape juice (25%) or red wine (0%) than in rats treated with saline solution (62.5%) or ethanol (75%).

**Conclusions:**

These results indicate that the cardioprotective response stimulated by resveratrol and grape products prevents the lethal cardiac arrhythmias in animal model of ischemia and reperfusion, supporting the idea that this treatment can be beneficial for prevention of severe cardiac arrhythmias in patients with ischemic heart disease.

## Introduction

Cardiovascular diseases remain the main cause of deaths in the worldwide being responsible for almost 18 million deaths annually, and about 80% of these deaths are attributed to ischemic heart diseases, such as acute myocardial infarction^1^. Although the reperfusion is the main treatment of these diseases, this process may aggravate the myocardial injuries produced during ischemia, generating severe and fatal arrhythmias due to collapse of cardiac excitation-contraction coupling (CECC) generated by ionic and bioenergetic deregulation in cardiac cells, such as ventricular arrhythmias and atrioventricular block^2^-^3^.The ventricular arrhythmias are tachyarrhythmias that originate in the heart’s ventricles, and include ventricular extrasystoles, ventricular tachycardia and ventricular fibrillation, the latter two being extremely severe forms of arrhythmia that can lead to death^4^. The causes of these forms of arrhythmias include heart disease or coronary artery disease^4^. This disease affects 2.5% of the world population and it is estimated that up to 10% of people over 75 are carriers^5^.

The first-degree atrioventricular block, or prolongation of the PR interval, corresponds to a disturbance in the cardiac electrical conduction, whose PR interval is prolonged for more than 0.20 seconds. The second-degree atrioventricular block is characterized by constant PR interval, before and after P wave blocked, with QRS complex large; and in the third-degree atrioventricular block there is no electrical communication between atria and ventricles or relationship between P waves and QRS complexes, which can lead to death^6^. Atrioventricular block has a prevalence of 0.65 to 1.1% in the general population, and an incidence of 0.13 for every 1,000 people, and increases the risk of atrial fibrillation^6^. The most common causes of these disturbances that severely compromise the CECC are cardiac diseases related to dysfunctions of the atrioventricular nodules, increased vagal tone, myocarditis, acute myocardial infarction, and also inflammatory and degenerative heart diseases^7^.

Several pharmacological and non-pharmacological strategies for the prevention of cardiac arrhythmias have been proposed to decrease the adverse effects and costs with these patients^8^. Among the various pharmacological prophylactic strategies with anti-arrhythmic potential, the resveratrol stands out, which can be found in high concentration in the grape peel and seed, as well as their manufactured products, especially red wine and grape juice^3^,^9^,^10^. In red wine, the concentration of resveratrol varies from 0.09 to 18 mg/L (average of 5 mg/L)^9^. The resveratrol (3,5,4’-trihydroxy-trans-stilbene) is a small molecule with a molecular weight of 228.246 g/mol with anti-inflammatory, antioxidant, hypoglycemic and antihypertensive and cardioprotective effects, which acts through multiple mechanisms of action, including the activation of the silent information regulator factor 2-related enzyme 1 (Sirt1), a nicotinamide adenine dinucleotide-dependent deacetylase involved in many physiological functions like oxidative stress^9^-^14^. Based on our recent studies, we have suggested that the combined molecular actions of resveratrol on Sirt1 and other cellular proteins involved in the regulation of functions of cardiac cells stimulates the cardioprotective response, attenuating or preventing the cytosolic and mitochondrial Ca^2+^ overload and bioenergetic collapse involved in cardiac arrhythmias caused by ischemia and reperfusion in cardiac cells^3^. However, there is insufficient information regarding the cardioprotective effects of resveratrol and grape products.

Since cardiovascular diseases represent the main cause of deaths in the worldwide, and cardiac arrhythmias are common in the general population, particularly in patients with ischemic heart diseases, our research group has invested efforts to develop new pharmacological strategies to treat these diseases. Thus, the present study aims to investigate the cardioprotective and antiarrhythmic effects produced by preventive treatment with resveratrol and grape products, such as grape juice and red wine, using an animal model of cardiac ischemia and reperfusion developed by our group.

## Methods

All experimental protocols were approved by Ethical Committee of the Universidade Federal de São Paulo (UNIFESP) – Process #2367271115.

The study enrolled adult male Wistar weight between 320 to 350 g with 12 to 14-week-old obtained from Center for the Development of Animal Models for Medicine and Biology (CEDEME/UNIFESP). These animals were maintained under standard conditions of nutrition, hydration, temperature, light and humidity, and in accordance to normalization approved were. All groups received treatment with diluted solutions daily and administered by intragastric administration orally (gavage) during 21 days prior to surgery to induction of CIR. Rats were submitted to protocol of cardiac ischemia and reperfusion after treatment with resveratrol (RES), grape juice (GJ) and red wine (RW) to evaluate the anti-arrhythmic effects of these treatments. Since the lethality of CIR+SS animals was previously known to be between 60-70%, a Fisher’s exact test sample size calculator was used to determine a total sample size ofat least 33 animals to detect a biologically relevant reduction of lethality to 20% (one-sided a = 0.05; power 0.8). This total was rounded up to 40 animals, divided in five experimental groups: 1) CIR group - treated with3.715 mL/kg/day of saline solution 0.9%, n = 8; 2) CIR+RES group - treated with RES 1 mg/kg/day, n = 8**;** 3) CIR+GJ group - treated with 3.715 mL/kg/day of Aurora^®^ whole GJ, n = 8; 4) CIR+ethanol (ET) group - treated with 3.715 mL/kg/day of 12.5% ethanol solution, n = 8; 5) CIR+RW group - treated with 3.715 mL/kg/day of RW (Malbec Wine, Valdorella®, containing 12.5% ethanol, n = 8). The doses administered to the animals corresponded, approximately, to 300 mL of GJ and RW consumed by humans, dose equivalent to1.3 mg/L of polyphenols, including the RES^15^,^16^.

### Surgical procedures for induction of cardiac ischemia and reperfusion (CIR)

Surgical procedures used for induction of CIR in rats were made in accordance with methodology previously described^17^-^19^. Rats were anesthetized with urethane (1.25 g/kg), and fixed in the supine position. After intubation (Jelco 14G, USA), rats were mechanically ventilated using a mechanic ventilator Insight model EFF 312 (Insight Equipamentos Científicos, Ribeirão Preto-SP, Brazil). After stabilization for15 min, thoracotomy was performed to place the vascular tourniquet (4/0 braided silk suture attached to a 10-mm micropoint reverse cutting needle, Ethicon K-890H, USA) around the left anterior descending coronary artery to induce ischemia. After of 10 min of cardiac ischemia, the tourniquet was removed to allow coronary recirculation for 75 min (cardiac reperfusion). The cardiac electrical activity in all groups studied was monitored by electrocardiogram (ECG) system using a method previously described^17^-^19^. ECG analysis was performed during 100 min of duration (stabilization for 15 min, cardiac ischemia for 10 min and cardiac reperfusion for 75 min). The ECG was recorded using a biopotential amplifier by means of needle electrodes placed subcutaneously on the limbs. Successful surgical obstruction of the coronary artery was validated by ECG alterations (increase in R wave and ST segment) caused by cardiac ischemia^17^-^19^. The body temperature was maintained at 37.5 ºC with a heated operating platform and appropriate heating lamps and was evaluated routinely via a rectal thermometer.

### Evaluation of cardiac activity by ECG analysis

The cardiac activity in rats submitted to CIR was evaluate by ECG in accordance with methodology previously described^17^-^19^. The ECG data were recorded using an acquisition system AqDados 7.02 (Lynx Tecnologia Ltda., Brazil), an acquisition system AqDados 7.02 (Lynx Tecnologia Ltda., Brazil), and analyzed using the software AqDAnalysis 7 (Lynx Tecnologia Ltda., Brazil). Using this software, the heart rates were evaluated, as well as incidence of ventricular arrhythmias (VA), atrioventricular block (AVB) and lethality (LET), in response to CIR. The ventricular fibrillation, torsades de pointes, and ventricular tachycardia parameters were considered only as VA.

### Statistical analysis

The incidence of VA, AVB, and LET were statistically evaluated using the Fisher’s exact test, and Prism 5.0 software (GraphPad, USA). Results were considered statistically significant when p < 0.05.

## Results

**Figure 1:**
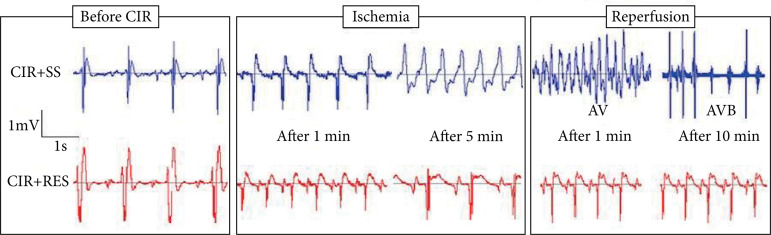
Typical record showing ECG obtained in animals from CIR+SS and CIR+RES groups. Note that the incidence of severe ventricular arrhythmias (VA) at the beginning of reperfusion evolved to atrioventricular block (AVB) after 10 min of reperfusion in CIR+SS, but not in CIR+RES group.

**Figure 2:**
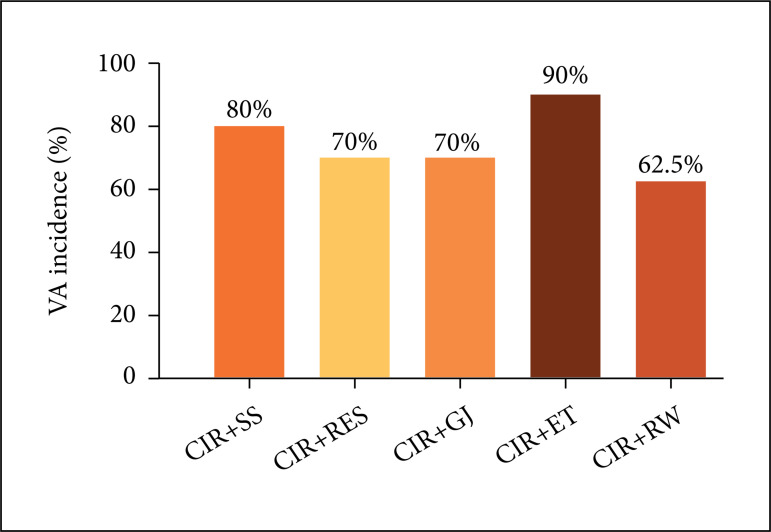
Incidence of the ventricular arrhythmias (VA) in the CIR+SS, CIR+RES, CIR+GJ, CIR+ET and CIR+RW groups. The results were expressed as mean, and analyzed by Fisher’s exact test (*p < 0.05).

**Figure 3:**
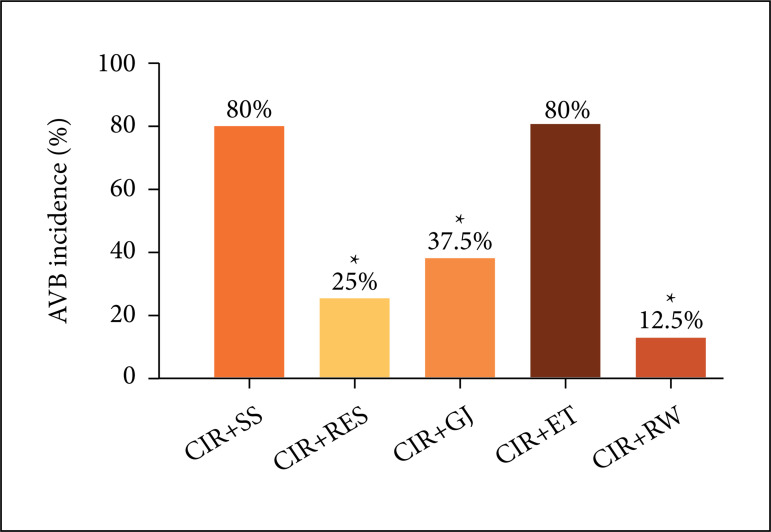
Incidence of the atrioventricular block (AVB) in the CIR+SS, CIR+RES, CIR+GJ, CIR+ET and CIR+RW groups. The results were expressed as mean, and analyzed by Fisher’s exact test (*p < 0.05).

In all groups studied, cardiac rhythm before CIR was maintained between 325 to 340 bpm, but varied significantly during ischemia and reperfusion, validating this animal CIR model to study of anti-arrhythmic effects produced by the preventive treatment with RES, GJ and RW. Fig. 1 shows the typical ECG record obtained in CIR+SS and CIR+RES. It was observed that the incidence of severe VA at the beginning of reperfusion evolved to AVB after 10 min of reperfusion in CIR+SS, but not in CIR+RES, CIR+GJ and CIR+RW groups. Fig. 2 shows that the incidence of VA in CIR+RES (70%), CIR+GJ (70%), CIR+RW (62.5%) groups was not statistically difference from CIR+SS (80%) and CIR+ET (90%). However, the incidence of AVB was significantly lower in CIR+RES (25%), CIR+GJ (37.5%), CIR+RW (12.5%) groups than in CIR+SS (80%) and CIR+ET (80%) control groups(Fig. 3), indicating the preventive treatment with RES, GJ and RW produced anti-arrhythmic effects in animal CIR model.

**Figure 4:**
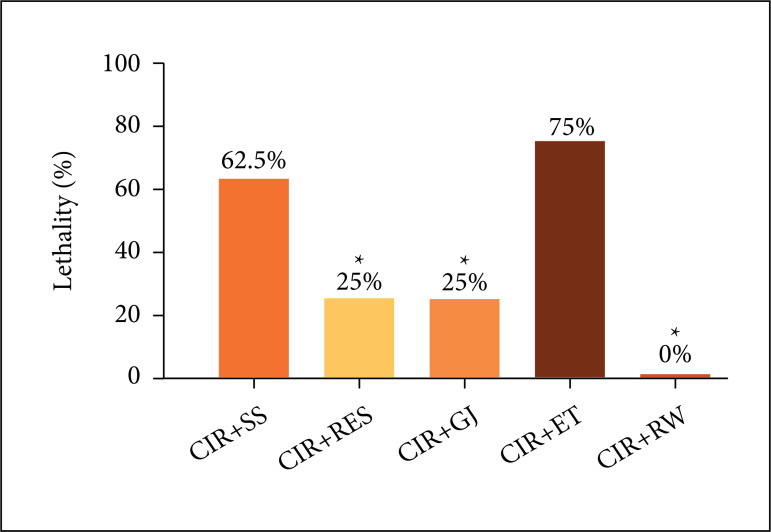
Incidence of the lethality (LET) in the in the CIR+SS, CIR+RES, CIR+GJ, CIR+ET and CIR+RW groups. The results were expressed as mean, and analyzed by Fisher’s exact test (*p < 0.05).

Fig. 4 shows that incidence of LET also was also significantly lower in CIR+RES (25%), CIR+GJ (25%), CIR+RW (0%) groups than in CIR+SS (62.5%) and CIR+ET (75%) controls groups. It is important note that LET was absence in CIR+RW group. These results confirm that preventive treatment with RES, GJ and RW produced cardioprotective effects in animal CIR model.

## Discussion

Severe cardiac arrhythmias following myocardial ischemia and reperfusion are the leading cause of mortality in patients with ischemic heart disease in the worldwide. It is well known that ischemia and reperfusion trigger a cascade of electrophysiological and biochemical events in cardiac cells, which can lead to myocardial damage and the occurrence of cardiac arrhythmias. Then, we have evaluated new cardioprotective strategies to treat the ischemic heart disease, especially reducing or preventing the cardiac arrhythmias. In the present study, we evaluated the antiarrhythmic effects produced by the 21-days oral treatment with resveratrol (1 mg/kg/day), and grape products (red wine and grape juice) in a dose equivalent of resveratrol, in animal model of cardiac ischemia and reperfusion. Our results showed that these treatments significantly reduced the incidence of atrioventricular block and lethality in animal model of cardiac ischemia and reperfusion, supporting the idea that this treatment can be beneficial in the prevention of severe cardiac arrhythmias in patients with ischemic heart disease.

Although pharmacological and non-pharmacological strategies are fundamental in the control and survival of patients with ischemic heart diseases, complementary and alternative therapies for the prevention of cardiac arrhythmias in this diseases have been used in the context of decreasing adverse effects and costs with these patients^8^, mainly after reports of the “French paradox” that suggested the consumption of grape products as beneficial for cardiovascular health due its cardioprotective anti-arrhythmic actions of resveratrol^20^-^27^. This recognition of the cardioprotective benefits of resveratrol initiated a range of studies in an attempt to uncover the molecular basis of resveratrol action.

The inconsistent findings between preclinical and clinical studies might be attributable to variable distribution of resveratrol to the desired tissues and cells. Pharmacokinetics studies have shown that the resveratrol is quickly absorbed by the gastrointestinal tract following oral administration with peak plasma concentration occurring within the first 30 minutes after low doses and 90 to 120 minutes after high doses^9^. These studies also showed that the resveratrol have significant bioavailability in cardiac tissue and high affinity for liver and kidneys, the latter is involved in its excretion^9^. At relatively low doses (1 to 5 mg/kg/day), resveratrol renders the heart resistant to ischemia and reperfusion injury by generating a survival signal in cardiac cells. Single doses of less than 1 g orally administered have not produced adverse effects. These effects such as diarrhea, nausea, abdominal pain, transient headache was only observed after oral administration of doses greater than 500 mg for 30 days^9^.

Several studies have shown that ingestion of products obtained from red grapes reduces oxidative stress and inflammatory processes in many organs and tissues, especially cardiovascular system^25^-^27^. It was showed that resveratrol has potent anti-inflammatory, hypolipidemic, platelet antiaggregant, vasodilator and antioxidant activity^11^,^21^,^24^. Cardiovascular activity of resveratrol and grape products has been observed *in vitro* and *in vivo* studies^25^-^27^. *In vivo* studies performed in animal model of ischemia and reperfusion showed that the treatment with resveratrol produces potent antiarrhythmic effects, reducing the incidence and duration of ventricular tachycardia and ventricular fibrillation^21^. These effects have been attributed to antioxidant activity of resveratrol as a consequence of inhibition of inducible nitric oxide synthase (iNOs) and modulation of production of endothelial nitric oxide synthase (eNOS) and neuronal nitric oxide synthase (nNOS), with significant reduction in the incidence of atrioventricular block and lethality^21^,^28^. These cardioprotective effects of resveratrol is also resultant from its stimulant action on the production of antioxidant enzymes, such as catalase, superoxide dismutase and glutathione peroxidase, consequently reducing the production of reactive oxygen^29^. It is important note that adstringinin (3.3’, 4’, 5-tetrahydroxystilbene), a resveratrol analogue with potent antioxidant activity and stimulant action on nitric oxide (NO) biosynthesis, also reduces the incidence of atrioventricular block and lethality in animal model of cardiac ischemia and reperfusion^30^.

The present study showed that the preventive treatment with resveratrol and grape products (grape juice and red wine) significantly reduced the incidence of atrioventricular block and lethality in animal model of cardiac ischemia and reperfusion, demonstrating the cardioprotective efficacy of this treatment. Our previous studies indicate that these treatments can attenuate or prevent the collapse of CECC caused by ischemia and reperfusion due to combination of multiple actions of resveratrol^3^. It is well established that this collapse is primarily triggered by ionic and bioenergetic deregulation caused by ischemia and reperfusion in cardiac cells^2^-^3^. During ischemia, the ionic deregulation in Ca^2+^ homeostasis in cardiac cells due mainly to inadequate functioning of Ca^2+^-ATPases and L-type voltage-activated Ca^2+^ channels (L-type Cav) results in cytosolic and mitochondrial Ca^2+^ overload, collapsing the mitochondrial function and ATP production^2^,^3^. During reperfusion, this Ca^2+^ overload is aggravated due to increased Ca^2+^ influx into cytosol through Na^+^/Ca^2+^ exchanger activity and the increment in formation of free radicals^2^,^3^. This last event produces oxidation of structural proteins and proteins involved in the respiratory chain, oxidation of pyridine nucleotides, changes in the permeability of internal mitochondrial membrane, decoupling of oxidative phosphorylation, and in consequence collapsing the mitochondrial ATP production and Ca^2+^ homeostasis in cardiac cells^2^,^3^.

*In vitro* studies using patch clamp methodology^31^-^34^ showed that the antiarrhythmic effects of resveratrol result from its inhibitory actions on the of L-type Cav combined with its excitatory actions on the slow-acting rectifier K^+^ channels (IKs). It is well known that IKs are important for the cardiac cell repolarization, without interfering with thefunction of rapid-acting rectifier K^+^ channels (IKr). In addition, resveratrol also selectively increased the K^+^ current mediated by ATP-sensitive K^+^ channels (K_ATP_) in cardiac cells, reducing excitability of cardiac cells. The actions of resveratrol on these channels prevent the CECC collapse, reducing the incidence of severe arrhythmias resulting from ischemia and reperfusion^35^.

 Our previous studies have shown that pharmacological blockade of cardiac L-type Cav constitutes an effective strategy to attenuate or prevent the cytosolic Ca2+ overload and collapse of the CECC, and consequently the cardiac arrhythmias resulting from myocardial ischemia and reperfusion^3^,^17^. We showed that blocking cardiac L-type Cav with nifedipine(1 and 30 mg/kg, IV, before cardiac I/R) significantly reduced the incidence of AVB (from 79% to 14%) and LET (from 70% to 14%), in animals subjected to CIR. Similarly, we showed in the present study that treatment with resveratrol(1 mg/kg/day, VO, for 21 days) significantly reduced the incidence of AVB (from 80% to 25%), and LET (from 62.5% to 25%), in animals subjected to CIR, due to its anti-arrhythmic action produced by the blocking of cardiac L-type Cav. This hypothesis was reinforced by the observation that treatment with grape products rich in resveratrol, such as red wine and grape juice, also significantly reduced the incidence of AVB (from 80% to 12.5% and 37.5%, respectively) and LET (from 62.5% to 25% and 0%, respectively), in animals subjected to CIR.

Another mechanism through which resveratrol suppresses ventricular arrhythmias involves an increase of the cardiac refractory period by inhibiting Na^+^ channels, and transient and sustained K^+^ currents^36^. The antiarrhythmic effects of resveratrol also result from its action on the late Na^+^ current (INaL) mediated by increase the activity of Na^+^-Ca^2+^ exchanger (NCX) currents, modifying the intracellular Ca^2+^diastolic concentration in ventricular myocytes^36^. These results are compatible with *in vivo* studies that demonstrated that treatment with resveratrol reduced ventricular arrhythmia and tachycardia induced by coronary artery ligation, with an increase in survival and suppression of cardiac remodeling in animals subjected to myocardial infarction, with electrocardiographic signals monitored using a telemetry transmitter implanted^35^.

In accordance with concepts and results published by our group^3^, there is a great interest in the role of cytosolic Ca^2+^ overload, mitochondrial dysfunction, oxidative stress on cardiac injury, such as atrial fibrillation by the modulation of signals that regulate the ionic channels that control the cardiac excitability by resveratrol^33^,^37^, or the regulation of the number of ectopic ventricular heartbeats by the combination of resveratrol with others drugs, for example 1,25-dihydroxyvitamin D (1,25 D)^38^. The arrhythmogenic activity similar to post-depolarization and delayed post-depolarization induced by cytosolic and mitochondrial Ca^2+^ overload and oxidative stress mediated by L-type Cav in ventricular cardiomyocytes by a mechanism dependent on calmodulin II (CaMKII) can be suppressedby the use of resveratrol^37^, just as atrial fibrillation induced bycoronary artery ligation in rabbits can be reduced by regulating ion channels via phosphoinositide 3-kinase (PI3K)/AKT/eNOS signaling pathway^39^. *Ex vivo* performed in tissue samples obtained from patients with atrial fibrillation showed that resveratrol was able to attenuate mitochondrial changes and activation of target genes by NF-kB by controlling the Ca^2+^ input current through the L-type Cav^40^. In addition to its anti-arrhythmic effect, resveratrol promotes a positive inotropic effect, similar to phosphodiesterase inhibitors 3-isobutylmethylxanthine (IBMX), and protects against the proarrhythmic effects of sympathomimetic drugs^41^.

Due to antiarrhythmic effects of resveratrol observed *in vitro* and *in vivo* models of ischemia and reperfusion, its preventive use has been evaluated under different pathological conditions that can lead to the appearance of arrhythmias. Arrhythmias secondary to cardiac disorders such as myocardial infarction are common in patients with diabetes mellitus^42^, and the combination of resveratrol and glibenclamide in diabetic animals decreases the frequency of arrhythmias during cardiac reperfusion associated with restoration of Kir6 protein expression and ion channels autonomy, such as K_ATP_ channels^43^. In humans with chagasic chronic cardiomyopathy, the oxidative stress can cause arrhythmias; however, resveratrol is able to reduce prolonged PR and QTc intervals, reversing sinus arrhythmias, atrial and atrioventricular conduction disorders via the AMPK pathway and reducing the production of reactive oxygen species^44^. In rheumatoid arthritis, an autoimmune disease, atrial remodeling occurs which can lead to the appearance of atrial fibrillation, but in collagen-induced arthritis rats, resveratrol was able to reduce the duration of atrial fibrillation episodes, in part by decreasing the production of IL-6, TNF-a and lower atrial remodeling^45^. Humans with very long chain acetyl-CoA dehydrogenase deficiency (VLCAD), an electron transfer flavoprotein-dependent enzyme located in the internal mitochondrial matrix, have a defect in the mitochondrial oxidation of long-chain fatty acids, leading to the development of hypertrophic heart disease and arrhythmias that can lead to death. The treatment of these patients involves the administration of glucose, a high caloric diet with medium chain triglycerides and supplementation with L-carnitine^46^. Using differentiated cardiomyocytes from pluripotent stem cells of a patient with VLCAD, it was found that resveratrol led to increased fatty acid oxidation by increasing mitochondrial biogenesis and control of cytosolic Ca^2+^ concentration, which could lead to better control of arrhythmias in these patients by consuming food containing resveratrol^47^.

In addition to cardiac activity, resveratrol also produces vasodilation by synthesis of NO^48^, but this phenomenon can also attribute to its ability to modulate Ca^2+^ concentration in the endothelial and vascular smooth muscle cells^49^-^53^. In these smooth cells, resveratrol inhibits intracellular Ca^2+^ release from the sarcoplasmic reticulum mediated by ryanodine (RyR) and inositol 1,4,5-triphosphate (IP_3_R) receptors, decreases the sensitivity of troponin-C to Ca^2+^, and promote an increase in sensitivity of cardiomyocytes^51^. We have proposed that the combination of pharmacological effects of resveratrol on the cardiomyocytes (antiarrhythmic actions) and coronary vascular cells (vasodilator actions) could decisively contribute to attenuate the myocardial injury and severe arrhythmias caused by long-term cardiac ischemia and reperfusion^3^.

These findings indicate that resveratrol is able to shorten the duration of cardiac arrhythmias, incidence of ventricular tachycardia and mortality caused by ischemia and reperfusion due to its multiple cardioprotective actions, especially by attenuation or prevention of production of reactive oxygen species (antioxidant activity), cytosolic Ca^2+^ overload and bioenergetic mitochondrial collapse in cardiac cells^3^,^11^,^14^.

## Conclusion

The results obtained in this study support the idea that the prophylactic use of resveratrol-containing grape-derived products prevents lethal cardiac arrhythmias in an animal model of ischemia and reperfusion, supporting the idea that this treatment can be similarly beneficial for prevention of severe cardiac arrhythmias in patients with ischemic heart disease. Further efforts are still required to broaden our understanding of how potential mechanisms, such as control of oxidative stress, intracellular Ca^2+^ homeostasis and mitochondrial dysfunction contribute towards the underlying mechanistic network and to narrow the knowledge gap between preclinical studies and human trials of resveratrol.
